# A Book on Cats

**Published:** 1895-05

**Authors:** 


### REVIEW.

Another valuable addition will be made shortly to veterinary literature in the announced forthcoming work entitled *A Book on Cats*, by Prof. R. S. Huidekoper. It will be full of general information of much worth to the busy practitioner, including the classification, varieties, care, diseases, and treatment, with profuse illustrations.

